# Splenic microRNA Expression Profiles and Integration Analyses Involved in Host Responses to *Salmonella enteritidis* Infection in Chickens

**DOI:** 10.3389/fcimb.2017.00377

**Published:** 2017-08-24

**Authors:** Peng Li, Wenlei Fan, Qinghe Li, Jie Wang, Ranran Liu, Nadia Everaert, Jie Liu, Yonghong Zhang, Maiqing Zheng, Huanxian Cui, Guiping Zhao, Jie Wen

**Affiliations:** ^1^Institute of Animal Science, Chinese Academy of Agricultural Sciences Beijing, China; ^2^Precision Livestock and Nutrition Unit, Gembloux Agro-Bio Tech, University of Liège Gembloux, Belgium; ^3^State Key Laboratory of Animal Nutrition Beijing, China; ^4^College of Animal Science, Jilin University Changchun, China

**Keywords:** MicroRNA, *Salmonella enteritidis*, next generation sequencing, chicken, clinical symptoms, spleen, miRNA-target genes

## Abstract

To understand the role of miRNAs in regulating genes involved in the host response to *Salmonella enteritidis* (SE) infection, next generation sequencing was applied to explore the altered splenic expression of microRNAs (miRNAs) and deregulated genes in specific-pathogen-free chickens. Birds were either infected or not (controls, C) and those challenged with SE were evaluated 24 h later and separated into two groups on the basis of the severity of clinical symptoms and blood load of SE: resistant (R, SE challenged-slight clinical symptoms and <10^5^ cfu / 10 μL), and susceptible (S, SE challenged-severe clinical symptoms and >10^7^ cfu/10 μL). Thirty-two differentially expressed (DE) miRNAs were identified in spleen, including 16 miRNAs between S and C, 13 between R and C, and 13 between S and R. Through integration analysis of DE miRNAs and mRNA, a total of 273 miRNA-target genes were identified. Functional annotation analysis showed that Apoptosis and NOD-like receptor signaling pathway and adaptive immune response were significantly enriched (*P* < 0.05). Interestingly, apoptosis pathway was significantly enriched in S vs. C, while NOD-like receptor pathway was enriched in R vs. C (*P* < 0.05). Two miRNAs, gga-miR-101-3p and gga-miR-155, in the hub positions of the miRNA-mRNA regulatory network, were identified as candidates potentially associated with SE infection. These 2 miRNAs directly repressed luciferase reporter gene activity via binding to 3′-untranslated regions of immune-related genes *IRF4* and *LRRC59*; over-expressed gga-miR-155 and interference gga-miR-101-3p in chicken HD11 macrophage cells significantly altered expression of their target genes and decreased the production of pro-inflammatory cytokines. These findings facilitate better understanding of the mechanisms of host resistance and susceptibility to SE infection in chickens.

## Introduction

*Salmonella enteritidis* (SE) is a Gram-negative enteric pathogen, infection with which does not cause significant disease or mortality, but birds can carry the bacteria for several weeks without presenting any clinical signs, thereby constituting an insidious risk for public health (Calenge et al., 2010; Barrow et al., 2012; Calenge and Beaumont, 2012). Although, Salmonella contamination can be significantly reduced using control measures in poultry, there was a considerable increase in reported Salmonella cases in the EU (European Food Safety Authority and European Centre for Disease Prevention and Control, 2016) and UK (Inns et al., 2015). SE also tends to be highly resistant to multiple antimicrobials, such as sulfamethoxazole-trimethoprim and nalidixic acid, which has the potential to complicate treatment of animal and human disease (DuPont and Steele, 1987; Goldman, 2004; Kuang et al., 2015). Therefore, to reduce economic losses in poultry production and to protect animal and human health, it is critical to understand the host immune response and mechanisms of resistance against SE infection.

MicroRNAs (miRNAs) have been identified as key regulators of gene expression at the post-transcriptional level. These small RNAs have been demonstrated to have important functions in a variety of biological processes including the cell cycle, differentiation, apoptosis, and pathogenesis (Ambros, 2004; Filipowicz et al., 2008; Krol et al., 2010; Yates et al., 2013). There are increasing evidences that the miRNAs play important roles in regulating the innate immune response induced by bacteria (Eulalio et al., 2012; Staedel and Darfeuille, 2013; Maudet et al., 2014; Das et al., 2016). Previous studies have shown that miRNAs, such as miR-146a, miR-155, and Let-7 and their targets are involved in the regulation of immune response to *Salmonella* or lipopolysaccharide infection in mice (O'Neill et al., 2011; Schulte et al., 2011; Eulalio et al., 2012) and swine (Bao et al., 2014, 2015; Yao et al., 2016a,b). For instance, few proteins (IRAK1, IRAK2, and TRAF6) within TLR signaling have been confirmed as direct targets of miR-146 (O'Neill et al., 2011); signal molecules MyD88, TAB2, SHIP1, and SOCS1 were targets of miR-155 (Eulalio et al., 2012); and cytokines IL-6 and IL-10 are targeted by Let-7 (Staedel and Darfeuille, 2013).

The role of miRNA in response to bacterial infection has also been investigated in chickens. Several miRNAs (gga-miR-125b-5p, gga-miR-34a-5p, gga-miR-1416-5p, and gga-miR-166) associated with SE infection were identified recently in laying chicken cecum by next generation sequencing (Wu et al., 2017). A novel splenic miRNA, gga-miR-429, involved in the host response to Avian pathogenic *Escherichia coli* (APEC) was also detected by deep sequencing (Jia et al., 2017). Despite these studies, there is still limited information about the function of miRNAs in the host response and resistance to *Salmonella* infection in chickens.

The spleen, as the body's major blood filter, plays a major role in detecting cell damage during *Salmonella* infection and in the pathogenic mechanisms of *Salmonella*. Further, increasing evidence suggests that the spleen plays a greater role in immune function in avian than in mammalian species, and is responsible for an immediate innate reaction after recognizing pathogens by filtering antigens from the blood (Smith and Hunt, 2004; Tiron and Vasilescu, 2008). Assessing changes in the expression of miRNAs and their targets in spleen on a genome-wide scale, therefore, could provide more comprehensive insight into the immune response to bacterial infection. The objectives of the present study were to identify the miRNAs and miRNA-regulated genes responsible for host resistance and susceptibility to SE infection using next generation sequencing on spleens from three groups of chickens: Controls (C, non-challenged, no detected SE in blood at 24 h, Resistant (R, SE-challenged, slight clinical symptoms, <10^5^ cfu/10 μL SE in blood), and Susceptible (S, SE-challenged, severe clinical symptoms, >10^7^ cfu/10 μL SE in blood) chickens. Subsequently, based on combined analysis of expression profiles of miRNA and potential target mRNA, the functional analysis and candidate miRNAs involved in the host response to SE infection were further characterized with the goal of better understanding the mechanisms of resistance and susceptibility to *Salmonella*.

## Materials and methods

### Ethics statement

All of the animal experiments were conducted in accordance with the Guidelines for Experimental Animals established by the Ministry of Science and Technology (Beijing, China). Animal experiments were approved by the Animal Management Committee (in charge of animal welfare issue) of the Institute of Animal Sciences, Chinese Academy of Agricultural Sciences (IAS-CAAS, Beijing, China). Ethical approval on animal survival was given by the animal ethics committee of IAS-CAAS (approval number: IASCAAS-AE20140615).

### Animals and sample collection

Specific-pathogen-free White Leghorn chickens were supplied by the Beijing Laboratory Animal Research Center (BLARC, Beijing, China) and were treated as described in previous studies (Li et al., 2010; Gou et al., 2012). In brief, the SPF chickens were raised in climate-controlled, fully enclosed isolation facilities at the experimental center of China Agriculture University (Beijing, China) under identical management conditions. At 3 d of age, a total of 150 SPF chickens were orally challenged with 1 ml PBS containing 10^8^ cfu of *S. enteritidis* (50041) and another 75 birds received 1 ml PBS as controls. Blood samples from each of 30 challenged and 15 control chickens were taken at 24 h post infection and birds were killed and the spleens were dissected, snap frozen and held at −80°C. Bacterial burden (expressed as cfu/10 μL blood) was determined indirectly by serovar-specific quantitative real-time PCR (qPCR), and along with clinical severity, was used to evaluate the resistance/susceptibility to SE challenge, as described in previous studies (Deng et al., 2008; Gou et al., 2012). 10 μL EDTA-anticoagulated blood was used for DNA extraction using MiniBEST Whole Blood Genomic DNA Extraction Kit (Takara, Code No. 9781) according to the manufacturer's instructions. Amplification was carried out in a total 25 μL reaction mixture, containing 0.6 μL of each primer (10 μM), 0.75 μL of dNTPs (10 mM), 1.25 U of ExTaq DNA Polymerase (Takara), 5.5 μL of 5 × PCR buffer (Mg^2+^), 0.8 μL of TaqMan probe (5 μM), and 2 μL of template, with deionized water to 25 μL. Each PCR consisted of a 5 min hot start at 95°C followed by 40 cycles of 30 s at 94°C, 30 s at 55°C, and a fluorescence read step. The probe (5′-FAM-TGCAGCGAGCATGTTCTGGAAAGC-TAMRA-3′) and primers set (forward primer, 5′-TCCCTGAATCTGAGAAAGAAAAACTC-3′; reverse primer, 5′-TTGATGTGGTTGGTTCGTCACT-3′) were designed from the *SdfI* gene (Gen-Bank Accession No. AF370707.1), as described in Gou et al. (2012). The qPCR assay was calibrated by relating threshold cycle (Ct) values to cfu, as determined by enumeration after plating serial dilutions of *S. enteritidis* and standard culture.

In this study, the clinical symptoms (diarrhea, drooping wings, and dying) and bacterial load of SE at 24 h after challenge were used together to discriminate susceptible (S, SE-challenged, slight clinical symptoms and >10^7^ cfu/10 μL blood) from resistant (R, SE-challenged, severe clinical symptoms and <10^5^ cfu/10 μL blood) birds. No SE was detected in the Controls (C). Total splenic RNA was extracted from three birds in each of the three groups, S, R, and C, using miRNeasy Mini Kit (Qiagen, Hilden, Germany) following the manufacturer's protocol. RNA was quantified using the NanoDrop ND-2000 spectrophotometer (NanoDrop Products, Wilmington, DE) and purity was assessed by Bioanalyzer 2100 and RNA 6000 Nano LabChip Kit (Agilent, Santa Clara, CA) with RNA Integrity Number (RIN) number >7.0. Total RNA was stored at −80°C until used.

### Small RNA sequencing and screening of the differentially expressed miRNAs

Total RNA of each sample (~1 μg) was used to prepare the miRNA sequencing library, which included the following steps: (1) 3′-adapter ligation with T4 RNA ligase 2 (truncated); (2) 5′-adapter ligation with T4 RNA ligase; (3) cDNA synthesis with an RT primer; (4) PCR amplification; and (5) extraction and purification of 120–140 bp PCR amplified fragments (corresponding to ~15–25 nt small RNAs) from polyacrylamide gels. An Agilent 2100 Bioanalyzer quantified the libraries, after which the samples were diluted to a final concentration of 8 pM and cluster generation was performed on the Illumina using TruSeq Small RNA Sample Prep Kits (Illumina, San Diego, CA), following the manufacturer's instructions. The 9 miRNA libraries were constructed and single-end sequenced (36 bp) on an Illumina Hiseq 2500 at the LC-BIO (Hangzhou, China) following the vendor's recommended protocol. The raw data of each sample was not <10 M reads. The raw reads were subjected to the Illumina Pipeline filter (Solexa v0.3), and then the dataset was further processed with ACGT101-miRv4.2 (LC Sciences, Houston, TX) to remove adapter dimers, junk, low complexity, common RNA families (rRNA, tRNA, snRNA, snoRNA) and repeats. Subsequently, the 18–25 nt length unique sequences were BLASTed to chicken precursors in miRBase 20.0 (Kozomara and Griffiths-Jones, 2014) (http://www.mirbase.org/) to detect known miRNAs and novel 3p- and 5p- derived miRNAs. One mismatch inside the sequence and length variation at both 3′ and 5′ ends were allowed in the alignments. The unique sequences were mapped to chicken mature miRNAs in hairpin arms recognized as known miRNAs, and mapped to the other arm of known chicken precursor hairpins opposite the annotated mature miRNA-containing arm considered to be novel 5p- or 3p-derived miRNAs. The remaining sequences were mapped to other selected species in miRBase 20.0 by BLAST search, and the mapped pre-miRNAs were further BLASTed against the chicken genomes to identify their genomic positions. The aforementioned miRNAs were considered to be known miRNAs. To identify the novel predicted miRNAs, the unmapped sequences were BLASTed against the chicken genome database, and the hairpin RNA structures comprising sequences were identified using RNAfold software (http://rna.tbi.univie.ac.at/cgi-bin/RNAWebSuite/RNAfold.cgi). Modified reads per million (RPM) reads was used to quantify the normalized reads, the formula was: Normalized Expression (NE) = Actual miRNA count/Total count of clean reads. MicroRNAs were regarded as being differentially expressed (DE) based on normalized deep-sequencing levels (with the exclusion of 3 RPM) in S, R and C groups, respectively. The DE miRNAs based on normalized counts were analyzed using Student *t*-tests (Huang et al., 2015; Li et al., 2016) according to the experimental design and the significance threshold was set as *P* < 0.05. The normalized read counts of some miRNAs were set to be 0.01 for further calculation if they had no reads in the library.

### Differentially expressed analysis of mRNA

Nine cDNA libraries were also constructed from splenic RNA (1 μg) of these same birds and sequenced by LC-BIO (Hangzhou, China) on an Illumina HiSeq 2500 platform and 125 bp paired end reads were generated. The raw reads were first processed through FastQC to obtain the clean data, by removing the reads that contain sequencing adapter contaminations or poly-N and the low quality reads, *Q*-values for which were <20. Some potential residual ribosome RNA data were also removed from the remaining data by alignment. Clean reads were then mapped to the Gallus gallus database using TopHat (Trapnell et al., 2009), and the mapped reads were assembled *de novo* using Cufflinks (Trapnell et al., 2010). Expression levels of mRNAs were quantified as fragments per kilobase of exon per million mapped reads (FPKM) using the Cufflinks package (Trapnell et al., 2010). Analysis of DE genes between the three groups of chickens was performed using the Cuffdiff with a *P* < 0.05 and |log_2_fold change| > 0.58.

### Prediction of DE miRNA targets, gene ontology (GO), and KEGG pathway analysis

Only target DE genes that were predicted by both TargetScan 6.2 and miRanda 3.3 for all of the DE miRNAs were considered further. Gene Ontology (GO) and KEGG pathway enrichment of target DE genes were analyzed by DAVID 6.8 (http://david.abcc.ncifcrf.gov/), which is based upon a Fisher Exact statistic methodology similar to that previously described (Huang et al., 2009). GO and KEGG results were filtered using *P* < 0.05.

### Correlation analysis of miRNA and mRNA

In order to build the miRNA-mRNA interaction network, the following method was used, as described in previous studies (Ye et al., 2016): A target gene was identified by the direction of change in a pairwise comparison, for example S to C, being the reverse of changes in the miRNAs. The miRNA-mRNAs interaction network was constructed using Cytoscape v2.8.3 software (http://www.cytoscape.org/).

### Mirna target validation

The pmiR-RB-Report™ (RiboBio, Guangzhou, China) including double luciferase reporter genes was used to test and validate the target sites for gga-miR-155 and gga-miR-101-3p. The 3′ UTR of *IRF4* and *LRRC59* containing gga-miR-101-3p and gga-miR-155 binding sites were amplified from chicken genomic DNA. The primers for PCR are provided, as follows: *IRF4*: GGCGGCTCGAGGATCCTCAGAATAAGTGTT (forward) and AATGCGGCCGCGTTAGAAG-TCCCTAGAAAA (reverse); and *LRRC59*: GGCGGCTCGAGATGCTACAGCAGAACTCGC (forward) and AATGCGGCCGCCAGACAAATTGATGCGAAA (reverse). All PCR products were cloned into the pmiR-Repor Vector using *Xho*l and *Not*I restriction enzymes. Luciferase reporter experiments were performed in 293T (human embryonic kidney) cells, obtained from ATCC. Cells were seeded in 96-well plates at a density of 5 × 10^4^ cells/well and cultured under routine conditions with 10% fetal bovine serum. When the cells reached 70 to 80% confluence, pmiR-3′ UTR (100 ng) was co-transfected with 50 nM of a negative control or a gga-miR-101-3p mimic (GenePharma, Shanghai, China) using 0.30 μL of FugeneHD (Promega, Madison, WI) according to the manufacturer's instructions. The relative luciferase activity was measured 48 h after transfection by the Dual-Glo Luciferase Assay System (Promega).

### Over-expressed gga-miR-155 and interference gga-miR-101-3p in chicken HD11 macrophage cells

To further validate the biological function of gga-miR-155 and gga-miR-101-3p in a chicken macrophage-like line HD11, 100 μM mimic (gga-miR-155), inhibitor (gga-miR-101-3p) and control oligos (gga-miR-NC) were transfected into HD11 cells using 12-well plates and TransIT®-2020 (Mirus Bio, Madison, WI) per the manufacturer's instructions. HD11 cells were grown at 37°C with 5% CO_2_ in RPMI-1640 medium that contained 10 mM HEPES, 1 mM sodium pyruvate, 1% glutamine, 1% MEM NEAA, 10% fetal bovine serum, and 5% chicken serum (all reagents from Gibco). After 36-h transfection, the cells were harvested using MiniBEST Universal RNA (Takara, Code No. 9767) to extract the total RNA. For the LPS exposure, macrophages were challenged with 1 mg/ml LPS and harvested at different times for RNA extraction. Cells with no stimulation were collected as the control, and each experiment had three biological replicates.

### Quantitative real-time PCR analysis

To validate and characterize the DE miRNA and DE transcripts identified via high-throughput sequencing, qPCR analyses were performed in an ABI 7500 Detection System (Applied Biosystems, Foster, CA). The miScript SYBR Green PCR kit (Qiagen, Valencia, CA) and PCR Master Mix (SYBR Green) Kit (Toyobo, Osaka, Japan) were used in qPCR to determine the abundance of mRNAs and miRNAs, using β-actin and U6 genes as reference genes, respectively. The relative mRNA and miRNA expression level was calculated using the 2^−ΔΔCt^ method (Livak and Schmittgen, 2001). All primers are described in Supplementary Tables S8, S9. Three independent replications were used for each assay and data are presented as means ± *SD*.

## Results

### miRNA profiles in the spleen of chickens

**Figure 1:**
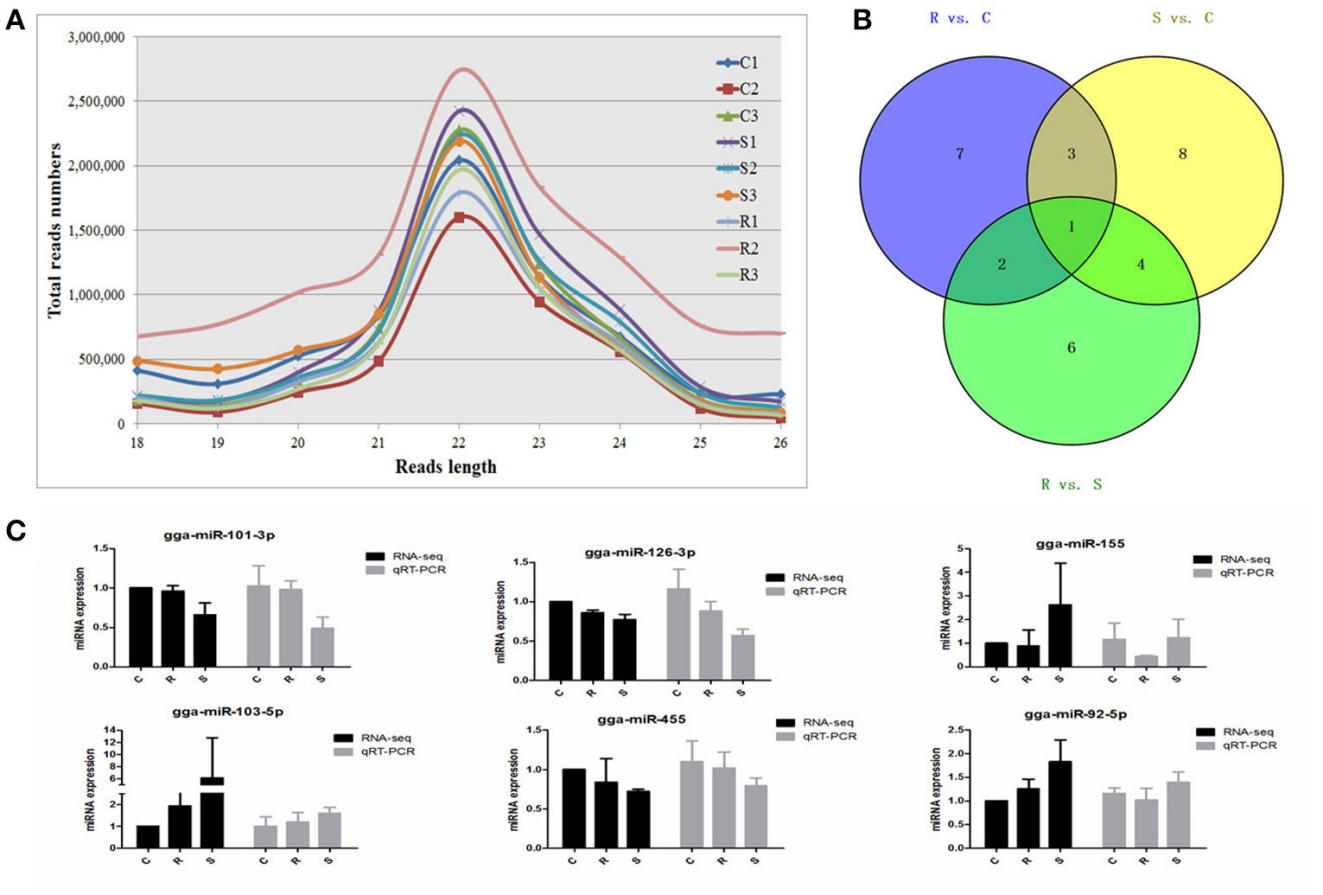
Different expression profiles of miRNAs among C, R, and S chickens. **(A)** Size distribution of sequenced small RNA reads. **(B)** Venn diagram demonstrates the overlap of differentially expressed (DE) miRNAs among the three groups; numbers are the DE miRNAs in each comparison. **(C)** Correspondence of miRNAs obtained by high-throughput sequencing and qPCR. C, Controls; R, Resistant; S, Susceptible.

An average of 6,348,747 high quality clean reads per miRNA sample, which represented 359,232 unique reads in the range of 18–26 nt in the nine libraries were obtained from splenic samples via next generation sequencing (Supplementary Table S1). These high-quality reads were mapped to chicken precursors in miRBase to identify known and novel miRNAs for further analysis. Low levels of large fragments, such as mRNA and rRNA, were also found, which indicated the high-quality and minimal degradation of RNA samples in the present study. For all nine samples, the distribution of the small RNA sequence length was mainly concentrated at 22 nt, followed by 23 and 21 nt (Figure 1A), which is consistent with the typical size range for Dicer-derived products and in agreement with most of the previous reports from other animal species.

A total of 2238 miRNAs, classified into five categories (Supplementary Table S2), were detected via BLAST in miRBase. After removing the less expressed miRNAs, i.e., the expression levels were <3 after the normalization of dataset (in at most 3 samples), 744 miRNAs were identified including 439 known chicken miRNAs, and 62 potentially novel miRNAs (defined as PC-3p or PC-5p) in chicken spleen after oral challenge with SE (Supplementary Table S2).

### Differential expression of miRNAs in response to *Salmonella* infection

A total of 32 miRNAs exhibited significantly different expression (DE) among the C, R and S groups. The results showed that, for S vs. C 16 DE miRNAs (7 up- and 9 down-regulated); for R vs. C 13 DE miRNAs were found (4 up- and 9 down-regulated) and 13 were found in the R vs. S comparison (10 up- and 3 down-regulated; Figure 1B, Table 1 and Supplementary Table S3). To validate the expression profiles from sequencing, 6 miRNAs were also examined by q-PCR (Figure 1C). Except for gga-miR-92-5p with a slight difference in the R group, the expression patterns of gga-miR-101-3p, gga-miR-126-3p, gga-miR-155, gga-miR-103-5p, and gga-miR-455 were comparable by both methods. The expression profiles from the deep sequencing were therefore considered as being reliable and appropriate for further analysis.

**Table 1:** Differential expression profile of splenic miRNAs among birds responding differently to SE infection.

**miR_name**	**Control group**			**Susceptible group**			**Resistant group**			**Fold change**		
	**C1**	**C2**	**C3**	**S1**	**S2**	**S3**	**R1**	**R2**	**R3**	**S vs. C**	**R vs. C**	**S vs. R**
gga-miR-30d	179,038	261,752	189,247	219,678	229,144	199,988	187,155	180,891	177,550	1.03	0.87	1.19
gga-miR-126-3p	108,338	110,264	129,549	90,066	85,231	90,663	94,410	98,790	107,131	0.76	0.86	0.89
gga-miR-101-3p	45,446	47,287	43,332	38,096	33,688	43,994	41,802	39,141	41,483	0.85	0.90	0.95
gga-miR-130b-3p	20,879	21,193	20,467	19,025	18,282	19,224	16,414	18,857	19,163	0.90	0.87	1.04
gga-miR-155	9,447	14,579	6,040	15,874	22,363	28,150	6,580	7,062	10,569	2.21	0.81	2.74
gga-miR-219b	7,612	9,004	6,773	8,128	7,685	7,064	6,775	6,554	6,625	0.98	0.85	1.15
gga-miR-455-5p	7,149	7,996	8,713	5,330	5,755	6,035	7,359	4,995	6,368	0.72	0.78	0.91
gga-miR-140-5p	3,053	3,135	4,058	2,908	2,964	2,592	3,344	3,602	3,629	0.83	1.03	0.80
gga-miR-181a-3p	2,214	2,963	1,926	3,352	3,973	3,186	2,460	2,659	2,985	1.48	1.14	1.30
gga-miR-181a-3p	2,214	2,963	1,926	3,352	3,973	3,186	2,460	2,659	2,985	1.48	1.14	1.30
gga-miR-1677-3p	1,904	1,900	1,955	1,757	1,862	1,804	1,702	1,588	1,424	0.94	0.82	1.15
gga-miR-1451-3p	233	225	271	160	279	241	197	155	194	0.93	0.75	1.25
gga-miR-137-3p	139	160	154	95	96	65	79	132	100	0.57	0.69	0.82
gga-miR-92-5p	137	192	120	271	253	262	158	219	178	1.75	1.24	1.42
gga-miR-100-3p	110	143	99	206	152	185	108	178	144	1.54	1.23	1.26
gga-miR-1781-3p	106	109	104	107	106	115	95	81	90	1.03	0.84	1.23
gga-miR-9-3p	77	110	86	61	25	45	151	28	39	0.48	0.80	0.60
gga-miR-1769-3p	61	22	40	59	114	129	76	91	73	2.46	1.97	1.26
gga-miR-3539	54	61	46	41	61	51	38	41	34	0.95	0.71	1.35
gga-miR-1306-5p	49	55	54	77	63	77	65	81	71	1.37	1.38	1.00
gga-miR-490-5p	44	30	38	25	1	11	56	20	37	0.33	1.01	0.33
gga-miR-1651-3p	44	47	39	48	44	56	41	37	34	1.14	0.87	1.32
gga-miR-1712-3p	29	38	30	33	37	31	19	25	28	1.04	0.75	1.40
gga-miR-6583-5p	19	20	11	2	4	11	8	11	23	0.34	0.85	0.40
gga-miR-1458	13	11	5	20	32	30	22	30	10	2.83	2.24	1.32
gga-mir-1662-p3	11	9	12	8	6	5	14	17	6	0.59	1.17	0.51
gga-miR-29c-5p	8	12	12	109	13	7	21	20	17	4.03	1.94	2.22
gga-miR-6701-3p	7	8	6	11	7	1	2	4	5	0.90	0.57	1.73
gga-miR-7460-3p	7	8	5	6	5	7	3	1	3	0.90	0.40	2.57
gga-miR-6575-5p	5	3	3	3	5	1	6	7	8	0.82	1.98	0.43
gga-miR-103-5p	2	1	5	8	9	5	1	3	5	2.75	1.47	2.44
gga-miR-1798-3p	1	4	1	11	2	1	13	15	16	2.33	7.72	0.32

*Three birds in each of the three groups were normalized to obtain the expression of transcripts per million using total clean reads count in this study. The P < 0.05 among C, R, and S was considered to be the differentially expressed miRNAs*.

The differences in splenic expression between the controls, resistant and susceptible birds were examined. Four miRNAs were significantly differently expressed in both S vs. C and R vs. C, and 5 in both S vs. C and S vs. R, as well as 3 in both R vs. C and R vs. S (Figure 1B, Table 1). Only 1 miRNA (gga-mir-1677) was significantly differently expressed in all three groups of birds (Figure 1B). Several miRNAs previously reported to be involved in immune responses such as miR-155, miR-9, miR-30, miR-126, and miR-29 families were identified. Also identified here were several new candidate miRNAs associated with SE infection, such as gga-miR-29c-5p (up-regulated, *P* = 0.01) and gga-miR-137-3p (down-regulated, *P* = 0.009).

### Differential expression of miRNA-targeted genes in response to SE infection

**Figure 2:**
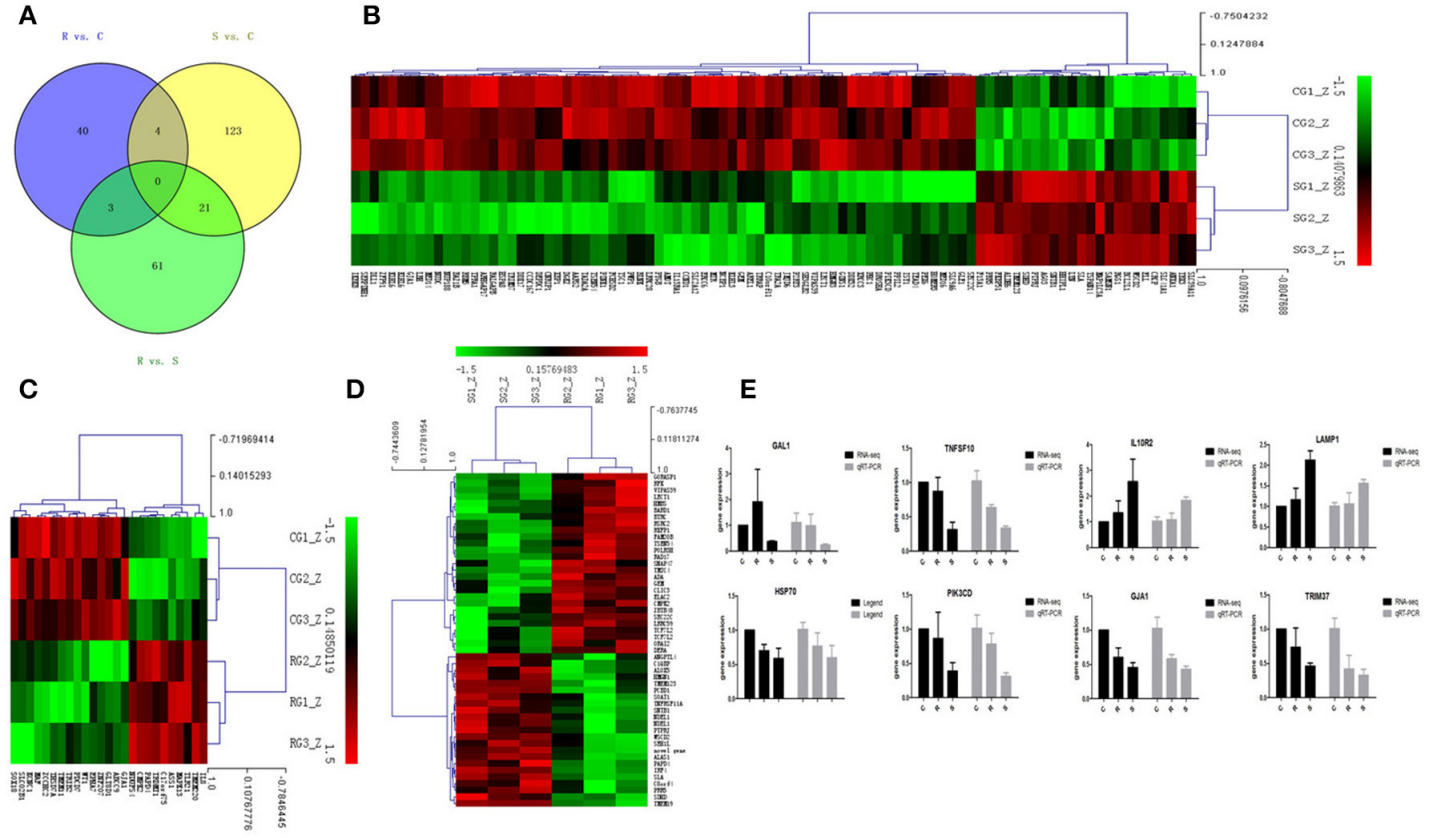
Differential expression of miRNAtargeted genes in response to SE infection. **(A)** Venn diagram demonstrates the overlap of targeted genes for the DE miRNAs among the three groups of chickens. Numbers in each section indicate the numbers of differently expressed miRNAs in the comparison. **(B–D)** The heat map of unique targets of DE miRNAs in S vs. C, R vs. C, and R vs. S, respectively. **(E)** Correspondence of the targeted genes for the DE miRNAs by high-throughput sequencing and qPCR. Data for each method were from the same samples of splenic tissues (C, R, and S chickens); TargetScan 6.2 and miRanda 3.3 were used to predict the miRNA targets and only targets predicted by both methods were used for further analysis. The heat map and clustering was constructed by Multi Experiment Viewer v4.8 using Row Z-Score (Murie et al., 2014) [(ΔΔCt–means)/SD] (Supplementary Table S7). In the figures, red represents up-regulation, green shows down-regulation, and black is no change.

In order to validate the roles of DE miRNAs in affecting expression of their target genes, mRNA in the same samples was also profiled by sequencing. Based on both TargetScan and miRanda systems, a total of 273 DE genes can be targeted by the 32 DE miRNAs in the three groups (Supplementary Table S4). As shown in Figure 2A, 148 (S vs. C), 40 (R vs. C), and 85 (R vs. S) DE miRNA-targeted genes were identified with fold change (FC) > 1.50 or < 0.67 and *P* < 0.05 (Supplementary Table S5). The heat map and hierarchical clustering demonstrated distinct profiles of the unique miRNA-targeted genes in the three comparisons (Figures 2B–D). Several immune-related genes were found to be significantly DE in spleen after challenge with SE. For example, the expression of *IL8, CXCR4*, and *IRF4* were significantly up-regulated following SE challenge (FC 5.21, 3.69, and 2.02, respectively). To validate the expression profiles from sequencing, transcript abundances of eight genes were measured by qPCR (Figure 2E); overall, there was good concordance between the two methods.

### Potential function analysis of DE miRNA targets

The ultimate function of miRNAs is at the level of the activity of target genes. In this study, functional annotation and pathway enrichment analysis of 273 target DE genes were performed using Gene Ontology (GO) and KEGG. Potential function analysis of these genes showed that 2 immune-related KEGG pathways and 1 biological process were significantly enriched (*P* < 0.05), including Apoptosis, NOD-like receptor signaling pathway, and adaptive immune response (GO:0002250) (Table 2). The present results suggest that the changed miRNAs may regulate these immune-related targets in chicken spleen during SE infection.

**Table 2:** Functional annotation and pathway enrichment analysis of all target genes were performed using GO and KEGG.

**Term**	**Description**	**Count**	**Percent (%)**	***P*-value**
gga04210	Apoptosis	5	2.1	1.50E-02
gga00562	Inositol phosphate metabolism	5	2.1	3.41E-02
gga04621	NOD-like receptor signaling pathway	4	1.7	3.74E-02
gga04630	Jak-STAT signaling pathway	6	2.6	5.66E-02
GO:0018149	Peptide cross-linking	4	1.7	1.54E-03
GO:0002250	Adaptive immune response	4	1.7	2.38E-02
GO:0001525	Angiogenesis	6	2.6	3.77E-02
GO:0000320	Re-entry into mitotic cell cycle	2	0.9	3.98E-02

*The potential targets of 32 differentially expressed miRNAs among C, R, and S chickens were used to identify enriched biological functions (P < 0.05). Only biological processes are listed*.

Potential functional analyses for host immune responses to SE infection between R and S chickens were further characterized, based on the target genes of significant DE miRNAs between these two groups and the controls. For S vs. C, 4 pathways were enriched (*P* < 0.05), viz. Apoptosis, Spliceosome, mTOR signaling pathway, Insulin signaling and Jak-STAT signaling pathway; 2 biological processes were significantly enriched (*P* < 0.05); regulation of inflammatory response and heart looping. In the R vs. C comparisons, NOD-like receptor signaling pathway was significantly enriched (*P* < 0.05); defense response to bacterium (GO:0042742), immune-related biological processes, was enriched but not significantly (*P* = 0.06). Except for regulation of proteasomal protein catabolic process (GO:0061136) being enriched (*P* < 0.05), no pathways were found when R was compared with S (Table 3). These results are consistent with the susceptible birds being more likely to exhibit apoptosis due to an inflammatory response, while the resistant birds showed more of an innate immune response to SE infection.

**Table 3:** GO and KEGG enrichment of unique miRNA target genes were analyzed between S vs. C, R vs. C, and S vs. R.

**Class**	**Term**	**Count**	**Percent (%)**	**P-Value**
S vs. C	Apoptosis	4	4.1	6.00E-03
	Spliceosome	4	4.1	3.81E-02
	Jak-STAT signaling pathway	4	4.1	4.70E-02
	Insulin signaling pathway	4	4.1	4.70E-02
	mTOR signaling pathway	3	3.1	4.70E-02
	GO:0050727, regulation of inflammatory response	3	3.1	2.80E-02
R vs. C	NOD-like receptor signaling pathway	2	14.3	4.60E-02
	GO:0042742, defense response to bacterium	2	8.3	6.00E-02
S vs. R	GO:0061136, regulation of proteasomal protein catabolic process	2	4.1	3.20E-02

*The results were filtered using P < 0.05*.

### miRNA-mRNA regulatory relationships in spleen after SE infection

**Figure 3:**
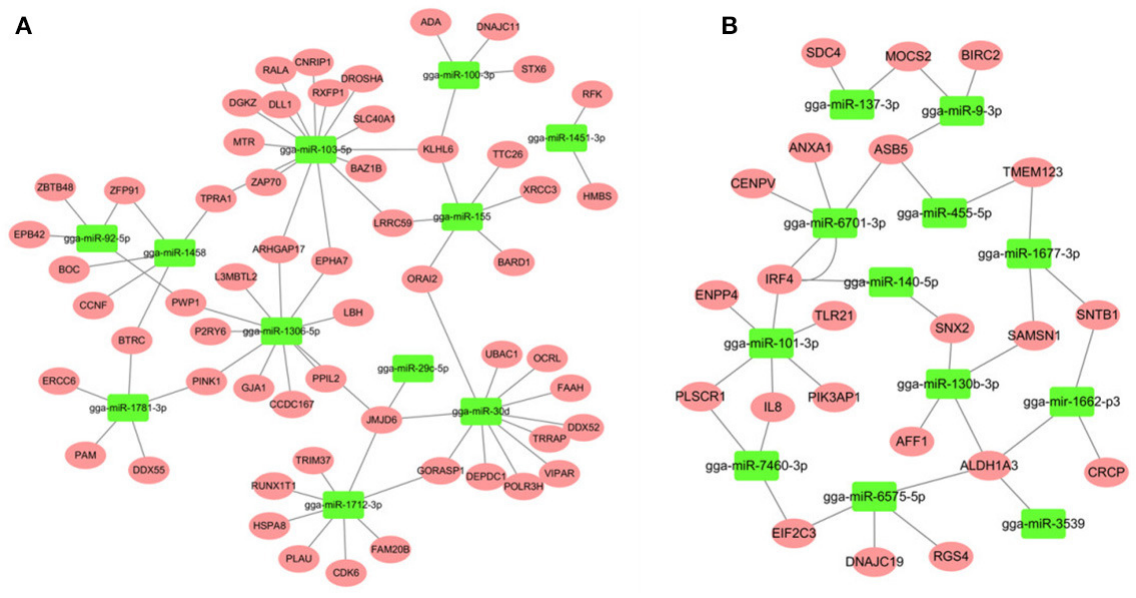
miRNA-mRNA interactions in spleen associated with SE infection. **(A)** miRNA-mRNA network among up-regulated miRNAs and down-regulated mRNAs **(B)** miRNA-mRNA network among down-regulated miRNAs and up-regulated mRNAs.

Most descriptions of miRNA function have focused on their roles as post-transcriptional regulators of target mRNAs. Based on the putative miRNA-mRNA regulatory pairs, it was found that 91 SE-related genes can be targeted by 29 of the 32 DE miRNAs (Supplementary Table S6). The potentially important interaction networks for immune-related miRNA-mRNA pairs are shown in Figure 3. The relative expression of innate/inflammatory marker genes such as *PIK3CD* was significantly up-regulated following SE infection. Some mRNAs are highly connected and regulated by multiple miRNAs. For example, *CXCR4* is involved in cytokine-cytokine receptor interaction and was identified as a potential target of gga-miR-155 and gga-miR-9-3p. *IFR4* was predicted to be regulated by gga-miR-30d and gga-miR-101-3p. *LRRC59* was predicted as a potential target of gga-miR-103-5p and gga-miR-155. One *Salmonella*-regulated miRNA of particular interest identified through the present study is gga-miR-101-3p. Although, the expression levels of miR-101-3p were relatively moderate, it is highly connected (>8 SE-related target genes) within the miRNA-mRNA network. These have not been previously reported to be associated with *Salmonella* infection, and are predicted here to regulate several immune-related genes.

### Validations of miRNA-mRNA interactions using gga-miR-101-3p-*IRF4* and gga-miR-155-*LRRC59* mimics

**Figure 4:**
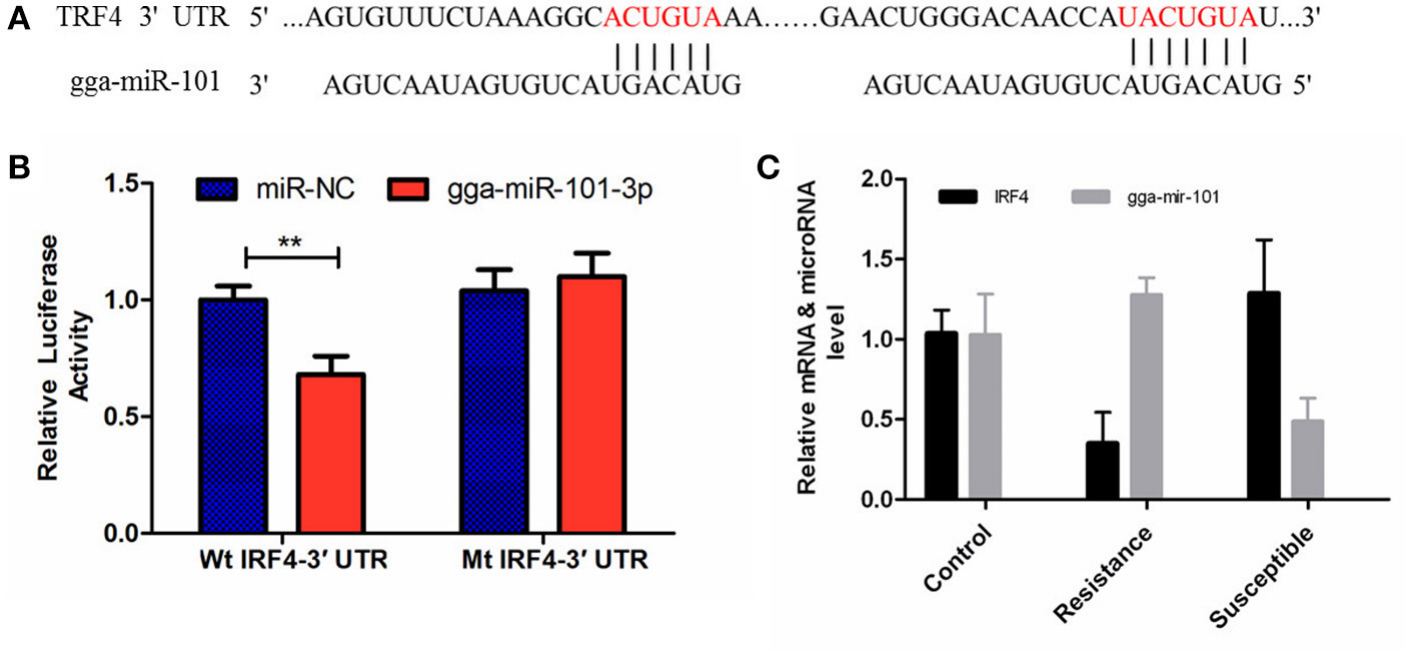
Regulation of *IRF4* by gga-miR-101-3p. **(A)** Predicted gga-miR-101-3p binding sites at distinct positions in *IRF4*; nucleotides of the gga-miR-101-3p seed region are in red. **(B)** Luciferase activity in 293T cells transfected with miRNA mimics and plasmids carrying the 3′UTR of *IRF4*. NC miRNA = negative control miRNA. **(C)** Expression change of *IRF4* and gga-miR-101-3p after infection. ^**^represents *P*-value < 0.01.

**Figure 5:**
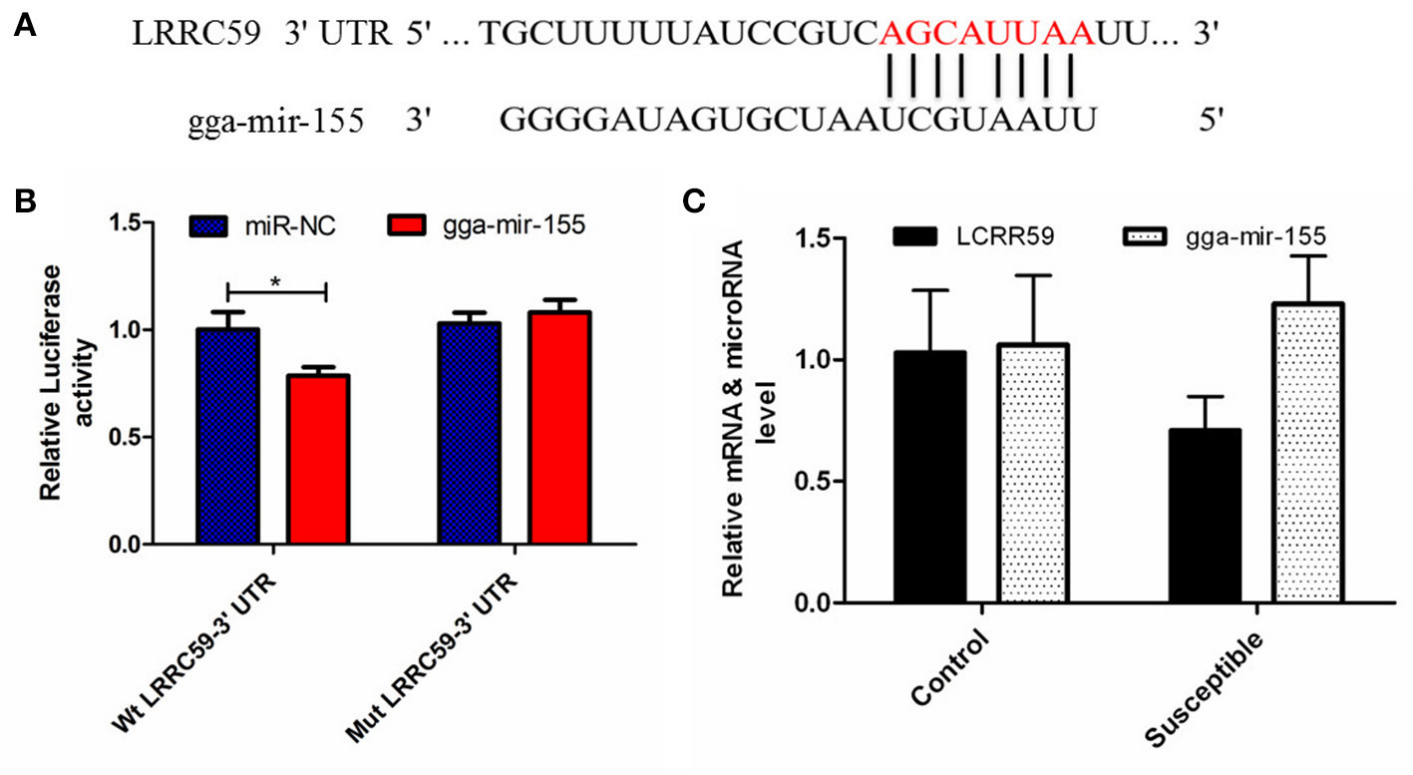
Regulation of *LRRC59* by gga-miR-155. **(A)** Predicted gga-miR-155 binding sites at distinct positions in *LRRC59*; nucleotides of the gga-miR-155 seed region are in red. **(B)** Luciferase activity in 293T cells transfected with miRNA mimics and plasmids carrying the 3′UTR of *LRRC59*. NC miRNA = negative control miRNA. **(C)** Expression change of *LRRC59* and gga-miR-155 after infection. ^*^represents *P*-value < 0.05.

The luciferase reporter gene system was used to validate the above-stated predicted interactions. The 3′ UTRs of *IRF4* and *LRRC59* were cloned into luciferase reporter plasmids to test gga-miR-101-3p and gga-miR-155 functions *in vitro*. Transfection with a gga-miR-101-3p mimic resulted in significant (*P* < 0.01) reduction in relative luciferase activity for *IRF4* plasmids (Figure 4), compared with negative control miRNA (random miRNA sequence) and a no-insert control. Similarly, transfections with mimics resulted in significant (*P* < 0.05) reduction in relative luciferase activity for *LRRC59* (Figure 5) compared with the negative miRNA and no-insert controls. These results indicate that similar responses are likely to be happening in the host during SE infection, that is, the down-regulation of gga-miR-101-3p may result in increased expression of *IRF4* during *Salmonella* infection, and up-regulation of gga-miR-155 may inhibit expression of *LRRC59*.

### Validations of biological function of gga-miR-155 and gga-miR-101-3p in chicken HD11 macrophage cells

**Figure 6:**
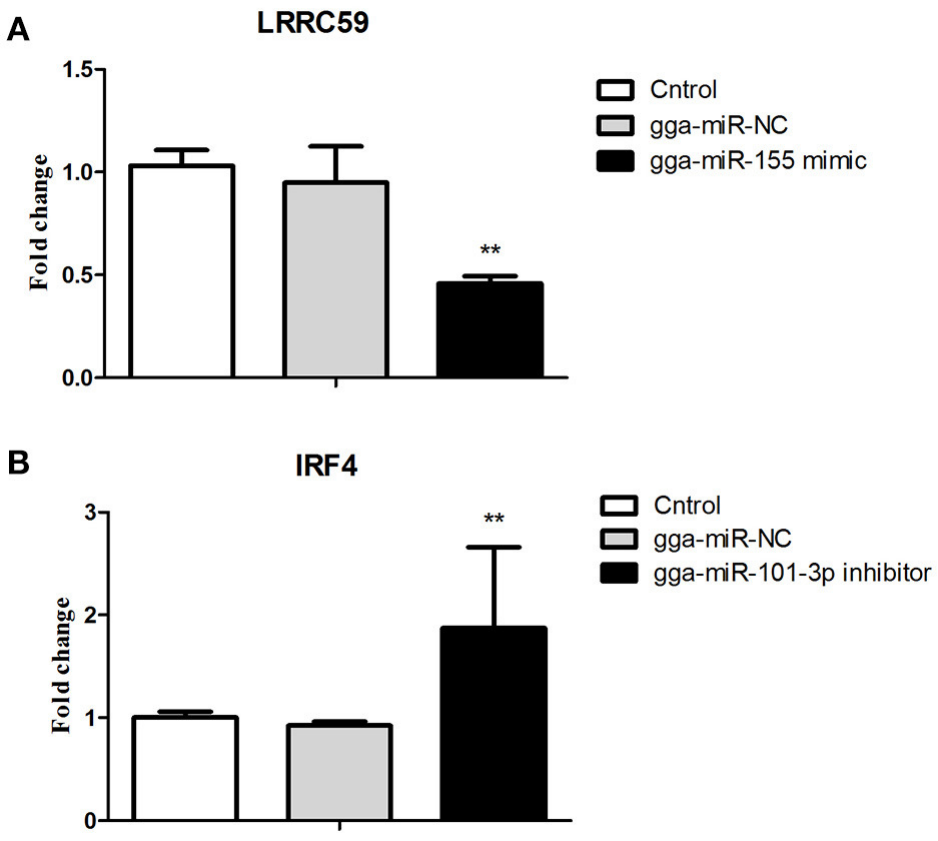
Validations of biological function of gga-miR-155 and gga-miR-101-3p in chicken HD11 macrophages. **(A)** gga-miR-155 mimic significantly repressed the mRNA expression of *LRRC59*. **(B)** gga-miR-101-3p inhibitor significantly promoted mRNA expression of *IRF4*. The fold-change values were calculated using the comparative 2^−ΔΔCT^. The *P*-values are indicated with asterisks when lower than 0.01 (^**^) when compared to control (non-transfected) and NC (gga-miR-NC).

**Figure 7:**
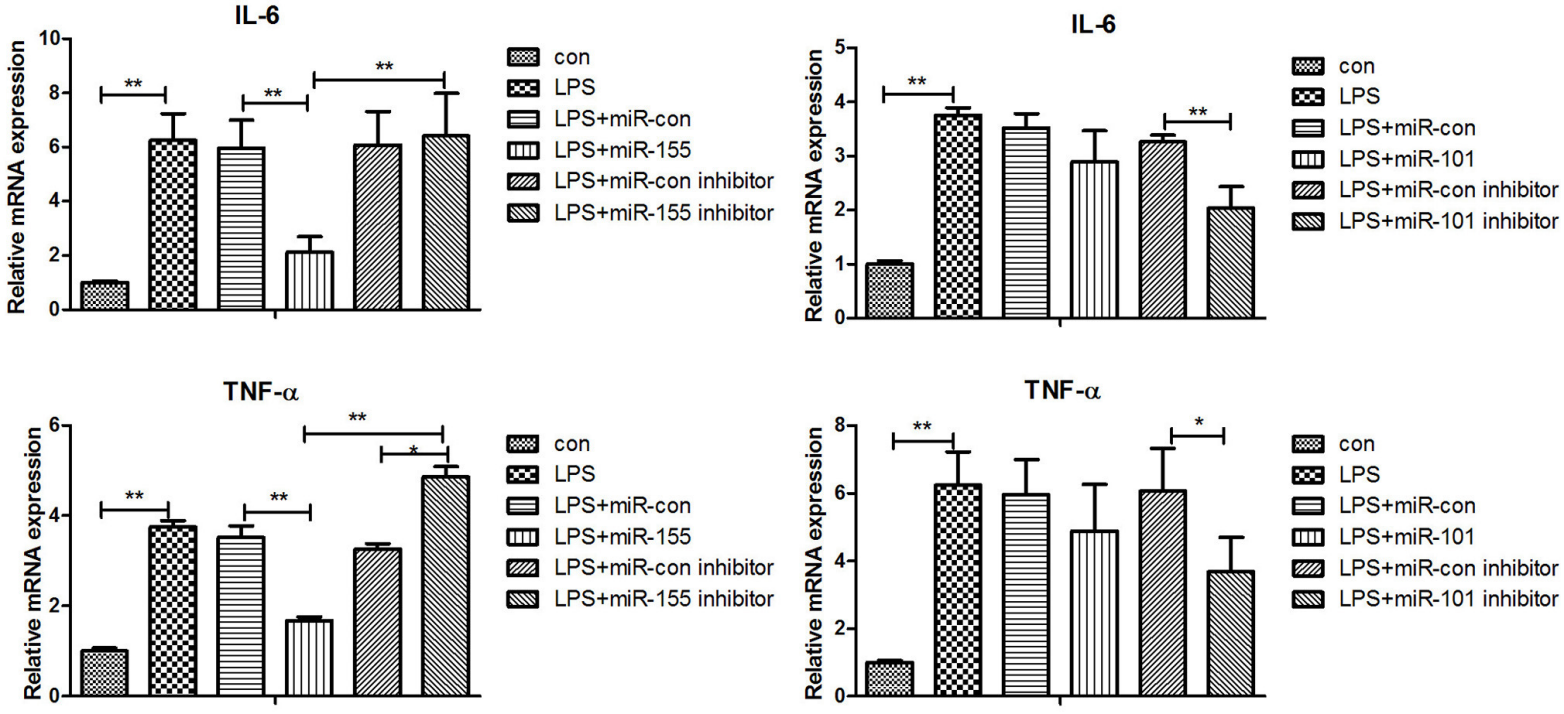
Gga-miR-155 and gga-miR-101-3p regulate expression of pro-inflammatory cytokine genes induced by LPS. mRNA expression of *IL-6* and *TNF*-α in chicken HD11 6 h after LPS treatment, or 24-h post transfection with miRNA control (50 nM), miRNA inhibitor control (100 nM), miRNA-155/101 (50 nM) and miRNA-155/101 inhibitor (100 nM) then the cells were stimulated with LPS (1 μg/mL) for 6 h. Relative transcript abundances of the genes were analyzed by qPCR. Data are presented as the mean ± SE from three independent experiments performed in triplicate. The *P*-values are indicated with asterisks when lower than 0.05 (^*^) or 0.01 (^**^) when compared to control.

After 36-h treatment with mimic, elevating gga-miR-155 significantly repressed the mRNA expression levels of *LRRC59* compared to the miR-NC and negative controls (*P* < 0.05); In contrast, after 36-h treatment with gga-miR-101-3p inhibitor, the mRNA expression levels of *IRF4* were significantly increased (*P* < 0.05) compared to the controls (Figure 6). In order to address the effect of miR-155 and miR-101 on the induction of pro-inflammatory cytokines in response to LPS, the expression levels of *IL-6* and *TNF*-α were measured in a macrophage inflammatory response model. The results showed that miR-155 overexpression markedly decreased the expression of *IL-6* and *TNF*-α compared with control miRNA or miR-155 inhibitor (Figure 7A; *P* < 0.01), while miR-101 knockdown significantly decreased the expression of *IL-6* and *TNF*-α compared with control miRNA inhibitor (Figure 7B; *P* < 0.05). These data demonstrate that gga-miR-155 and gga-miR-101 could regulate the production of pro-inflammatory cytokines, IL-6 and TNF-a, which may play a negative role in response to LPS stimulation in chickens.

## Discussion

MiRNAs are important regulators of innate and adaptive immunity (Sonkoly et al., 2008; O'Neill et al., 2011; Olivieri et al., 2013) but their specific roles in regulating the responses to *Salmonella* infection in chicken are incompletely understood. It is necessary, therefore, to identify and characterize the critical miRNAs in the chicken immune response to *Salmonella* with the aim of understanding pathogenesis, improving animal welfare, reducing losses in poultry production and in keeping food safe. Here, next generation sequencing was used to detect differences in splenic expression profiles of miRNAs in chickens challenged with SE. A total of 439 known and 62 potentially novel miRNAs were detected, including those expressed at low levels such as gga-miR-7460 (normalized average 7 and 2 reads for C and R, respectively). Through DEG analysis, 32 miRNAs were found to be differentially expressed among C, R and S groups, representing differences between both infected and non-infected animals and heavy and light bacterial burdens resulting from a single-dose infection with SE. For these miRNAs, gga-miR-155 had the most abundant expression and it was significantly up-regulated in susceptible chickens (both S vs. C and S vs. R). Similarly, gga-miR-92-5p was highly up-regulated in resistant birds (R vs. C and R vs. S). Another highly expressed miRNA, gga-miR-1306-5p, was increased in both R and S compared with C, but with no significant difference between R and S. This suggests that these miRNAs in spleen might be involved as components of the immune response to SE. These results of the present study also suggested that deep sequencing technology has utility in the discovery of functional miRNAs, including those expressed at low levels, in the SE pathogenic processes. Also in this study, three groups were defined to increase the power of detecting miRNA DE, according to the severity of clinical symptoms and host carrier-state level (quantified as cfu/ unit volume of blood), allowing comparisons to be made between birds demonstrating resistance or vulnerability to SE, in addition to simply comparing challenged and non-challenged birds. This is clearly a useful approach to identify the candidate genes involved to host resistance to SE. The present study of splenic miRNA and mRNA profiles from chickens after *Salmonella* challenge has identified differential expression of several miRNAs linked to immune responses, including miR-155, miR-9, miR-30 which have been reported previously and several miRNAs, such as miR-101-3p and miR-130b-3p, which were shown here to be associated with the immune response to infection with SE.

It is useful to predict miRNA function and construct regulation networks by the prediction of their targets and annotation of their biological function. Two immune-related KEGG pathways and one biological process were significantly enriched: Apoptosis, NOD-like receptor signaling pathway, and adaptive immune response. Interestingly, apoptosis pathway and regulation of inflammatory response were mainly enriched in the S vs. C comparison, while NOD-like receptor pathway and defense response to bacterium were enriched in the R vs. C comparison. These results indicated that miRNAs may play different regulatory roles associated with the extent of pathogen load in response to infection with SE, that is, between the susceptible and resistant birds.

Through the integration of miRNA and mRNA expression data and miRNA-RNA target prediction analysis, a number of putative miRNA-mRNA interactions were identified. Since hub nodes have been found to play important roles in many networks (He and Zhang, 2006), the presence of hub miRNAs was sought and, several were identified including gga-miR-155 and gga-miR-101-3p (Figure 3). It has been shown that miR-155 is involved in the TLRs signaling pathway and play important roles in the innate immune response (Quinn and O'Neill, 2011; Elton et al., 2013; Li and Shi, 2013). In contrast, gga-miR-101-3p has not been previously linked to *Salmonella* infection; the present finding in chicken spleen is novel.

The leucine-rich repeat (LRR) containing protein (LRRC) 59/p34 is a type II transmembrane protein with a short C-terminal domain facing the lumen of the endoplasmic reticulum (ER) and four LRRs and coiled-coil domain facing the cytosol. LRRC59 resides in the ER and nuclear membrane, and is reported to have the function of nuclear import of fibroblast growth factor (Skjerpen et al., 2002) and CIP2A (Pallai et al., 2015) at the nuclear membrane. Although, little is known about the function of *LRRC59*, it is becoming clear that this family of proteins, could have far-reaching effects on the immune response. A recent study showed that LRRC59 dependent trafficking of nucleic acid-sensing TLRs might be beneficial for augmentation of antimicrobial immune responses from the endoplasmic reticulum via association with Uncoordinated 93 homolog B1 (UNC93B1) (Tatematsu et al., 2015). MiR-155 has been reported to play important roles in both innate and adaptive immunity in mammals. Its expression is up-regulated after activation of the innate response in murine macrophages by lipopolysaccharide, CpG and poly (I:C) and it can down-regulate these signaling pathways by targeting key signaling molecules (Elton et al., 2013; Li and Shi, 2013; Olivieri et al., 2013; Maudet et al., 2014). In the current study, gga-miR-155 was significantly induced by SE infection, which was consistent with the above mammalian studies. Interestingly, the expression of gga-mir-155 was significantly higher in the S chickens compared with R birds. The expression of *LRRC59* here was significantly down-regulated (*P* = 0.02) in S vs. R chickens. The *in vitro* experiment showed that gga-miR-155 directly repressed the expression of *LRRC59*; In addition miR-155 overexpression markedly decreased the expression of *IL-6* and *TNF*-α compared with control miRNA or miR-155 inhibitor (*P* < 0.01). These results indicate that gga-mir-155 could target gene *LRRC59* and then suppress the production of pro-inflammatory cytokines in response to LPS challenge.

Interferon regulatory factor 4 (IRF4) is a transcription factor of the IRF family that plays pivotal roles in the negative regulation of TLR signaling. Several previous studies have demonstrated that, in macrophages, IRF4 negatively regulates the production of pro-inflammatory cytokines such as IL-6 and TNF-α in response to TLR ligands (Honma et al., 2005; Negishi et al., 2005). IRF4 interacts with MyD88 and acts as a negative regulator of TLR signaling by competing with IRF5 (Negishi et al., 2005). It is well recognized that the innate immune response is critical to controlling the replication of pathogenic microorganisms, especially in young mammals and birds (Kawai and Akira, 2011; Keestra et al., 2013). In this study, the expression of *IRF4* was significantly up-regulated in S compared to uninfected C birds (FC = 1.92, *P* = 0.03) and in S vs. R comparisons (FC = 2.62, *P* < 0.01). The expression of gga-miR-101-3p was significantly down-regulated in S vs. C (*P* < 0.01). In addition, gga-miR-101-3p directly inhibited *IRF4* expression and miR-101-KO significantly decreased the expression of *IL-6* and *TNF*-α compared with control miRNA inhibitor (*P* < 0.05).

Based on the foregoing observations and interpretations, it is reasonable to propose that gga-miR-155 and gga-miR-101-3p contribute to SE-induced pathogenesis and regulate the production of pro-inflammatory cytokines through directly down-regulating *LRRC59* and up-regulating *IRF4* genes, respectively.

In conclusion, this paper presents the first characterization of the splenic miRNA expression profile of the chicken in response to SE infection. A total of 32 DE miRNAs were identified among three phenotypic groups of chickens consisting of non-challenged controls, birds that were resistant to challenge with SE, and those that were susceptible to SE with heavy pathogen loads at 24 h after infection. Through integration analysis of DE miRNAs and DE mRNAs, a total of 273 miRNAs-targeted genes were identified. Immune-related Apoptosis and NOD-like receptor signaling pathway were found to be significantly enriched. Two miRNAs, gga-miR-155 and gga-miR-101-3p, could directly alter the expression of target *IRF4* and *LRRC59* and regulate the production of pro-inflammatory cytokines, respectively. These investigations indicate that miRNAs in spleen play a major role in the SE infection process. The findings will facilitate understanding resistance and susceptibility to *Salmonella* infection through miRNA-induced systems, provide guidance on potential vaccine targets, and may assist breeding for genetic resistance to SE in poultry.

## Author contributions

PL and WF performed experiments and data analysis and draft writing; QL and RL revised the manuscript and contributed to experiments and data analysis. JWa contributed to the animal study and data analysis. NE contributed to experimental design and revised the manuscript. JL, YZ, MZ, and HC contributed to animal experiments and data analysis and interpretation. GZ and JWe designed the experiments and supervised and coordinated the study. All authors reviewed the manuscript.

## Data availability statement

The raw sequence data reported in this paper have been deposited in the Genome Sequence Archive (Wang et al., 2017) in BIG Data Center Members (2017), Beijing Institute of Genomics (BIG), Chinese Academy of Sciences, under accession numbers CRA000315 that are publicly accessible at http://bigd.big.ac.cn/gsa.

### Conflict of interest statement

The authors declare that the research was conducted in the absence of any commercial or financial relationships that could be construed as a potential conflict of interest.
