# Supplementary material for: Splenic microRNA Expression Profiles and Integration Analyses Involved in Host Responses to Salmonella enteritidis Infection in Chickens
Source: Front Cell Infect Microbiol. 2017 Aug 24;7:377. doi: 10.3389/fcimb.2017.00377 (PMC5573731; doi:10.3389/fcimb.2017.00377)
Supplement: Supplementary file 2 [file DataSheet2.docx]

Supplementary Material

**MicroRNA expression profiles and integration analyses involved in host responses to *Salmonella enteritidis* infection in chickens**

Peng Li, Wenlei Fan, Qinghe Li, Jie Wang, Ranran Liu, Nadia Everaert, Jie Liu, Yonghong Zhang , Maiqing Zheng, Huanxian Cui, Guiping Zhao*, Jie Wen*

*** Correspondence:** Corresponding Author: [wenjie@iascaas.net.cn](mailto:wenjie@iascaas.net.cn) and [zhaoguiping@caas.cn](mailto:zhaoguiping@caas.cn)

# Supplementary Tables

**Table S1. Overview of reads from raw data to cleaned sequences.**

**Table S2. Summary of known and predicted miRNA in this study.**

**Table S3. List of all differential expression of miRNAs in S vs. C, R vs. C and R vs. S, respectively.**

**Table S4. List of all differential expression miRNA targets predicted using both TargetScan and miRanda..**

**Table S5. List of all differential expression of miRNA­targeted genes in S vs. C, R vs. C and R vs. S, respectively**

**Table S6. List of all miRNA-mRNA interactions identified here and their associated gene ontology terms.**

**Table S7. The raw Z-Score of miRNA­targeted genes which were used to generate the heat map.**

**Table S8. qPCR primers for miRNA­targeted genes.**

**Table S9. miRNA-specific primers for qPCR.**
